# Psychological assessment of the cognitive development of children with IVF: The experience of neuropsychological approach

**DOI:** 10.1192/j.eurpsy.2021.1108

**Published:** 2021-08-13

**Authors:** A. Sergienko, N. Zvereva, M. Zvereva

**Affiliations:** 1 Clinical Psychology, Federal State Budgetary Scientific Institution Mental Health Research Center, Moscow, Russian Federation; 2 Clinical Psychology, Federal State Budgetary Scientific Institution Mental Health Research Center; MSUPE, Moscow, Russian Federation

**Keywords:** IVF, Children, cognitive development, neuropsychology

## Abstract

**Introduction:**

The first stage of interdisciplinary studying (EEG, IQ, immunology, psychology, neuropsychology) of cognitive development of children with IVF, conducting at the Mental Health Research Center (Moscow, Russia) is presenting. A small number of studies analyzing the cognitive development of children in a wide age range, determined the design of this study.

**Objectives:**

Assessment of the capabilities of the neuropsychological approach in qualifying the cognitive development of children with IVF.

**Methods:**

20 children aged 7 to 15 years old, born with IVF, studying in school. Neuropsychological diagnosis according to A.R. Luria-L.S.Tsvetkova. All participants signed voluntary consent to participate in the study.

**Results:**

1. Neuropsychological approach and methods of neuropsychological diagnostics are effective in qualifying the neurocognitive development of children with IVF 2. The overwhelming majority of the examined children (90%) had energy factor dysfunction (at the level of brain stem structures in 65%, at the level of diencephalic structures in 82%, combined disorders at both levels in 52%) 3. Regulatory inhibitory control (impulse control - suppression of the dominant reaction) was impaired (functionally unformed) in 58% of the subjects 4. A gross violation of the kinetic factor was found in 46% of the examined children and adolescents IVF 5. The development of speech and visual memory is variable.

**Conclusions:**

The conclusions are preliminary and require testing on a wider sample of children born because of IVF and other assisted reproductive technologies. It is necessary to study the functional state of other neuropsychological factors, to expand the number of participants.

